# Bronchoscopic Lung Volume Reduction

**DOI:** 10.1155/2011/610802

**Published:** 2010-12-09

**Authors:** Armin Ernst, Devanand Anantham

**Affiliations:** ^1^Pulmonary, Critical Care and Sleep Medicine, St. Elizabeth Medical Center, VP Thoracic Disease and Critical Care Service Line, Caritas Christi Health Care, Seton 6 East, 736 Cambridge Street, Brighton, MA 02135, USA; ^2^Department of Respiratory and Critical Care Medicine, Singapore General Hospital, Outram Road, Singapore 169608

## Abstract

The application of lung volume reduction surgery in clinical practice is limited by high postoperative morbidity and stringent selection criteria. This has been the impetus for the development of bronchoscopic approaches to lung volume reduction. A range of different techniques such as endobronchial blockers, airway bypass, endobronchial valves, thermal vapor ablation, biological sealants, and airway implants have been employed on both homogeneous as well as heterogeneous emphysema. The currently available data on efficacy of bronchoscopic lung volume reduction are not conclusive and subjective benefit in dyspnoea scores is a more frequent finding than improvements on spirometry or exercise tolerance. Safety data are more promising with rare procedure-related mortality, few serious complications, and short hospital length of stay. The field of bronchoscopic lung volume reduction continues to evolve as ongoing prospective randomized trials build on earlier feasibility data to clarify the true efficacy of such techniques.

## 1. Introduction

Bronchoscopic techniques for the management of emphysema have evolved from the success of surgical treatment. Lung volume reduction surgery involves the removal of 20% to 30% of each lung and targets the most emphysematous segments. Patients with heterogeneous upper lobe emphysema and a low baseline exercise capacity have been identified as a subgroup within Chronic Obstructive Pulmonary Disease (COPD), in whom even mortality benefits can be achieved along with improvements in exercise capacity and quality of life [1]. This subgroup excludes patients with FEV_1_ and diffusion capacity <20% because of high surgical risks.

Air trapping and neuromechanical dissociation, that is, the disparity between effort and ventilatory output, are the mechanistic links that are being targeted by lung volume reduction [2]. Both airway narrowing and loss of elastic recoil cause expiratory air flow limitation in COPD. During exercise, any increase in respiratory rate shortens expiratory time and can result in air trapping. This air trapping reduces inspiratory capacity and limits ventilation. As a result of the restriction on inspiratory capacity, any increase in minute ventilation can then only be achieved by increasing respiratory rate further that in turn results in a vicious cycle of more dynamic air trapping.

Lung volume reduction attempts to correct loss of elastic recoil by reducing the volume of the most damaged lung segments and allowing the remaining less damaged tissues to resize. By eliminating parts of emphysematous lung with the longest expiratory time constants and removing dead space, dynamic air trapping is reduced and exercise capacity can be increased. The operating length of respiratory muscles is also normalized by restoring the normal dimensions of both the chest wall and the diaphragm. 

Increased short-term mortality of approximately 5% and postoperative morbidity are major limitations of surgical lung volume reduction [1]. The reported rate of intraoperative complications is 9% and postoperative complications is 58.7% with elevated risks for reintubation (21.8%), arrthymias (18.6%), pneumonia (18.2%), readmission to the intensive care unit (11.7%), and tracheotomy (8.2%) [3]. Air leaks of a median duration of 7 days have also been reported in up to 90% of patients [4]. In the National Emphysema Treatment Trial (NETT) study, up to 28% of patients were hospitalized or living in a nursing home/rehabilitation facility at 1 month after surgery [1]. Unfortunately, the price of all this postoperative morbidity and mortality does not guarantee benefits. Only 30% of patients in the most favorable subgroup of COPD with upper lobe disease and low baseline exercise tolerance derived a clinically significant improvement in exercise capacity of more than 10 watts and 48% registered a greater than 8-point decrease in the St. George's Respiratory Questionnaire at 24 months [1].

The extremely restrictive selection criteria coupled with the relatively high morbidity have been the likely reasons for the decrease in patients undergoing surgical lung volume reduction since the publication of the NETT data [5]. This situation persists in the United States despite established criteria for Medicare coverage of surgery. These factors have been the incentive for developing less invasive endoscopic modalities. Bronchoscopic lung volume reduction has pursued various approaches using a range of modalities such as blockers, stents, valves, thermal vapor ablation, sealants, and implants (Table 1). The physiological basis of each modality is not identical and in some cases distinct from conventional lung volume reduction surgery. The ideal indications also differ with airway bypass stents targeting homogenous emphysema, while valves and thermal vapor ablation target heterogeneous emphysema. Biological sealants and endoscopic coil implants have been used in both homogenous and heterogeneous emphysema.

## 2. Endobronchial Blockers

Endobronchial blockers affect resorption atelectasis by occluding airways leading to emphysematous lung segments. Initially silicone vascular balloons filled with radio opaque contrast were inserted before the advent of custom-built stainless steel stents with a central occlusive sponge [6]. However, the high rate of endobronchial blocker migration, postobstructive pneumonia, and the need for repeat endoscopic procedures have limited further development of this technique [6].

## 3. Airway Bypass Stents

Airway bypass involves the creation of extra-anatomic bronchial fenestrations to deflate emphysematous lung parenchyma. This technique relies on the presence of collateral ventilation which is the ventilation of alveoli through anatomic channels that bypass the airways. These channels include interalveolar pores, accessory bronchiole-alveolar connections, accessory respiratory bronchioles, and interlobar pathways across fissures [7]. Although collateral ventilation plays an insignificant role in normal lungs, in emphysema where there is increased airflow resistance, severely obstructed lung segments are ventilated by these channels. The degree of collateral ventilation may also correlate with the extent of homogeneity of emphysema [8]. In endoscopic airway bypass, the newly created low resistance bronchial fenestrations allow trapped air to escape by bypassing high resistance obstructed airways. Distal emphysematous lung segments are drained via collateral ventilation through these fenestrations and lung compliance is improved by reductions in dead space [9]. This improvement in lung compliance occurs without any actual change in pulmonary elastic properties. Instead inspiratory capacity available for gas exchange is increased by reduction in air trapping [10].

Current airway bypass procedures are performed on patients with homogenous emphysema. There are 3 steps that are performed via flexible bronchoscopy: identification of an area of the segmental bronchi that is free from blood vessels using a Doppler probe, fenestration of the airways, and placement of a paclitaxel eluting stent. Paclitaxel is a mitotic inhibitor that prevents granulation tissue from obstructing the stent.

The current published data on airway bypass is a multicenter, open labeled study on 35 patients with homogenous emphysema who had bypass stents placed in both lungs [11]. A median of 8 stents with a range of 2 to 12 were inserted [11]. Efficacy data at 6 months was limited. Mean residual volume was reduced by 400 ml and dyspnoea as measured on a modified Medical Research Council scale dropped by 0.5 points [11]. No significant changes were recorded on spirometry, 6-minute walk, and St. George's Respiratory Questionnaire. 

One death from hemoptysis has been reported with airway bypass procedures [11]. Data and safety monitoring board review of the fatal hemoptysis had the following recommendations which were incorporated into subsequent procedures: placement of an endobronchial balloon blocker in the main bronchus as well as Doppler rescanning between fenestration creation and stent deployment. Failure to implant stents is another possible intraoperative problem because of either excessive peribronchial blood vessels or markedly increased airway wall thickness [11]. Postprocedure complications occurred in 59% of cases with COPD exacerbation in 32%, pneumomediastinum in 5% and respiratory infection in 27% [11]. At followup bronchoscopy 6 months later, 69% of stents remained patent. Granulation tissue, radial traction by the surrounding airways and secretions have been identified as possible causes of stent occlusion [12].

## 4. Endobronchial Valves

Endobronchial valves are designed to exclude the worst affected emphysematous regions from ventilation and consequently reduce dynamic air trapping. If segmental or lobar resorption atelectasis can be induced, a physiological effect similar to surgical lung volume reduction is also expected. Therefore, patients with heterogeneous emphysema are ideal candidates for endobronchial valve therapy. Valves allow one-way flow of secretions and air out of an occluded pulmonary segment during expiration but prevent any distal flow during inspiration [13]. Currently 2 different endobronchial valve designs are being studied: duckbill and umbrella-shaped valves.

The duckbill valves are supported by a nitinol self-expanding, tubular mesh that is covered with a silicone membrane to form a seal between the valve and the bronchial wall. One-way exit of distal air and mucous is facilitated by the central duckbill. The valves are mounted on to a loading catheter and deployed via the working channel of a flexible bronchoscope. Deployment involves 2 stages: an endoscopic measurement gauge is used to size the bronchial diameter before a valve of the equivalent size is chosen. The loading catheter with the chosen valve is advanced to the target airway and the valve is deployed by using an actuation handle. A perfectly positioned endobronchial valve can be visualized on bronchoscopy with the proximal edge sitting flush with the carina of the segmental bifurcation. There is now emerging case report data on silicone duckbill valves that are inserted via rigid bronchoscopy [14]. These valves are easy to insert or remove and obviate the need for any special loading catheters.

Umbrella-shaped valves have a nitinol framework comprising 6 support struts. These struts are covered by a polyurethane membrane that seals the airways. Air and mucous can escape proximally around the edges of the membrane but distal flow is limited. A central rod enables removal or repositioning of the valve. After the airway orifice is sized with a calibrated water-filled balloon, these valves are deployed via a catheter loader through the working channel of a flexible bronchoscope.

The efficacy data for endobronchial valve therapy are inconclusive because all published studies have been open labeled trials with no control arms and have relatively short 3 to 6 month followup. Current data on endobronchial valves describe procedures that have been performed in one or both lungs with the intent of either lobar or nonlobar exclusion. Furthermore, there is a wide variation in number of valves (4–11) deployed [13, 15–17]. These variables confound efficacy comparisons between valves and make it nearly impossible to combine data. Nevertheless, in all studies the physiological measures of improvements have been limited. Despite being the most studied bronchoscopic lung volume reduction technique, patient numbers are still relatively small (*n* = 127 for duckbill valves and *n* = 98 for umbrella-shaped valves in the published literature up till the publication of the VENT study) [13, 15, 16, 18]. Large standard deviations have also led many investigators to report data as minimal clinically significant differences, that is, >15% as compared to preprocedure levels. Such differences in FEV_1_ were observed in 18 to 46% of patients at 3 months. On 6-minute-walk testing 11% to 55% had a minimal clinically significant difference after endobronchial valve therapy [13, 15, 19]. The reported improvements in subjective parameters such as dyspnoea and quality of life have been more impressive. Up to 85% of patients reported a marked improvement in dyspnoea at 30 days and significant improvements on the St. George's Respiratory Questionnaire is a frequent finding [16, 19–21]. Whether endobronchial valve therapy should target both lungs like surgery or just one lung remains controversial. The duckbill valve data suggest that unilateral therapy is better while the umbrella-shaped valve data favor bilateral treatment [13, 16, 22]. The reason for this conflicting finding remains unknown and is a subject of continued research.

Lobar atelectasis was not achieved in the majority of patients even with a lobar exclusion approach whereby all the bronchi to a target lobe were occluded with valves [20, 21]. In most series, less than 25% of cases reported lobar atelectasis [13, 15, 23]. Incompleteness of fissures between lobes of the lung and collateral ventilation accounts for this finding. However, the greatest benefits appear to be found in patients who actually do develop such target lobe atelectasis because of favorable changes in chest wall dimensions [18, 24]. Endobronchial approaches to measure resistance of collateral ventilation have been developed by using pressure measurements in balloon occluded lung segments [18]. Patients who developed atelectasis recorded collateral ventilation resistance of several orders of magnitude higher than those who did not. This suggests that endobronchial collateral ventilation assessment can accurately identify patients who will develop atelectasis after endobronchial valve therapy [18]. Future, bronchoscopic lung volume reduction studies may need to incorporate such assessment techniques to preferentially select patients with high collateral ventilation resistance as they are more likely to reap greater benefits from treatment. 

The symptomatic benefits that have been observed with endobronchial valves may be explained by other physiological mechanisms as well [22, 25]. By occluding airways, valves increase resistance to airflow such that air is diverted to other relatively less emphysematous parts of the lung resulting in reduced air trapping [25, 26]. By excluding the most diseased parts of the lung from ventilation, physiological dead space is reduced even in the absence of any atelectasis [25]. 

Current evidence suggests that endobronchial valve therapy is safer than surgical lung volume reduction. The only death directly attributable to endobronchial valve placement was due to postobstructive pneumonia [13]. Hospital length of stay has been short with the majority of patients discharged within 2 to 4 days [13, 15, 17, 19, 23]. Commonly reported postprocedure complications are COPD exacerbations (5% to 20%) and pneumothorax (7% to 11%) [13, 15, 19, 23, 25]. There appears to be a positive association between the development of lobar atelectasis and pneumothorax occurrence [16]. Pleural adhesions, rapidly expanding bullae in nontargeted lobes and an ex vacuo phenomenon may account for this [16, 25]. Targeting the lingular segment during left upper lobe therapy also appears to increase the risk of left-sided pneumothoraces [16]. Despite the single reported mortality, the frequency of postobstructive pneumonia is rare. 

The technical challenges facing endsocopists include difficulty of placement of endobronchial valves in segmental bronchi of the upper lobes. If moderate sedation is used instead of general anesthesia, respiratory movements and cough can impede accurate placement and adjustment [13, 19]. Proximal hyperplastic granulation tissue has also been observed on followup bronchoscopy. This granulation tissue may occlude the valves and prevent future removal should the need arise [15, 19]. Incidentally, endobronchial valves have found an unrelated therapeutic indication in the management of persistent pulmonary air leaks and have managed to affect either resolution or reduction in pneumothorax in >90% of cases [27].

## 5. Thermal Vapor Ablation

Controlled doses of steam, when delivered to a segmental airway, can produce an inflammatory response that results in lung volume reduction. The advantage of this technique is that no prosthesis needs to be inserted. The degree of collateral ventilation is also not an issue because the treatment works at the level of the lung parenchyma. A nonreusable 2 mm vapor catheter is inserted via flexible bronchoscopy to the target airways. On the vapor catheter, there is a distal occlusion balloon that isolates the lung segment. A precise dose of steam generated by an electronically controlled pressure vessel is then delivered to the isolated airways [28]. 

In a safety and feasibility trial, 11 patients with heterogeneous emphysema were treated unilaterally with a dose of 5 calories per gram of lung tissue [28]. Lung tissue weight was estimated from CT volume and density analysis. There were no recorded improvements in spirometry but mean St. George's Respiratory Questionnaire scores dropped 15.3 units from 64.4 to 49.1 over 6 months. Adverse events included COPD exacerbations in 4 patients and 2 episodes of pneumonitis [28]. Given this proof of concept, efficacy and safety data are now being sought.

## 6. Biological Lung Volume Reduction

Biological agents aim to reduce lung volume by sealing off the most emphysematous areas. The rapidly polymerizing sealant is designed to work at the alveolar level rather than in the airways. The mechanism of action involves resorption atelectasis from airway occlusion, subsequent airspace inflammation, and then remodeling. This remodeling will lead to scarring-induced contraction of lung parenchyma, and functional lung volume reduction can be expected within 6 to 8 weeks [29]. The sealant causes blockage of interalveolar as well as bronchiolar-alveolar collateral channels and negates the effects of collateral ventilation. This technology aims to achieve benefits by actual reductions in dead space that is physiologically similar to surgery [30]. 

After identifying a target region, the distal airways in this segment are collapsed by wedging the flexible bronchoscope in the bronchial orifice and applying suction. Initially, 10 ml of a primer (5000 U porcine trypsin) is instilled via the bronchoscope working channel to deactivate surfactant and promote detachment of epithelial cells [30]. After 2 minutes, the primer is suctioned out and then 10 ml of a cell culture media is used for wash out. A dual-lumen catheter is inserted through the working channel and advanced to within 2 cm of the distal end of the bronchoscope. A fibrinogen suspension and thrombin solution are then instilled simultaneously such that they are mixed in the lumen of the airways distal to the catheter tip. Then 60 ml of air is injected through the bronchoscope working channel to push the reagents distally. As the fibrinogen and thrombin mix, they polymerize into a hydogel within 30 seconds [29]. 

Bilateral therapy was instituted in 50 patients with upper lobe predominant emphysema in a phase 2 multicenter trial [31]. Eight bronchopulmonary subsegments were occluded in either a single endoscopic session or in a staged manner. Serious adverse events were documented in 4 patients due to aspiration, pneumonia, pulmonary embolism, and a fall related to analgesia. However, there were no documented fatalities. Procedure-related COPD exacerbations were observed in 22%. Postoperative leucocytosis, fever, or malaise occurred in 89% over first 24 hours [31]. 

The primary endpoint of a significant reduction in RV/TLC at 3 months was met [31]. This reduction was sustained at 6 months in only the patients receiving high dose therapy (20 ml per subsegment compared to 10 ml). Minimal clinically important differences at 6 months were also identified in both groups in FEV_1_ (38% to 44%); 6-minute walk (27%), and St. George's Respiratory Questionnaire (32% to 46%). Spirometric improvements and radiological evidence of remodeling were greater in patients who received high dose treatment [31]. 

Similar efficacy findings and safety profile were found in 25 patients with homogenous emphysema in a subsequent study [32]. However, despite having homogeneous emphysema, these patients had poorer perfusion to either the upper lobes or the apical segments of the lower lobes as evidenced by quantitative scintigraphy scanning. Predictably, the most damaged lobes identified by CT scanning that also had the poorest perfusion were targeted. Again the 20 ml high dose therapy had better efficacy results without any increase in complications.

Biological lung volume reduction appears safe and a dose-dependent response has been identified. In order to achieve the 20%–30% of lung volume that is removed in surgery, up to 12 subsegments may need to be sealed in future efficacy studies [30]. Unlike endobronchial valves, this therapy is not easily reversible and long-term followup data is critical. There are also concerns that atelectasis may diminish with time because of biodegradation of the hydrogel [26]. An interesting parallel development has been the bronchoscopic injection of autologous blood and fibrinogen into an emphysematous bulla to affect similar volume reduction [33].

## 7. Airway Implants

Airway implants such as nitinol coils of 10 to 20 cm in length have been designed for use in patients with either homogeneous or heterogeneous emphysema. These implants which are straight when housed in a delivery catheter, coil up on deployment and tether the lung. The coils are inserted under fluoroscopic guidance with each insertion taking less than 2 minutes. Preliminary safety data on 11 patients have shown no evidence of pneumothorax or severe adverse events [34]. Maximal reduction in lung volume occurred between 2 to 4 weeks after implantation and there is some suggestion of improvements in spirometry, exercise capacity, and quality of life [34]. The trend was for greater improvement in patients with heterogeneous emphysema [34]. Despite these promising results, concerns remain that the coils by distorting bronchi will cause bronchiectasis and by kinking pulmonary vessels will cause pulmonary infarcts. This technique remains very much in its infancy of development.

## 8. Conclusion

Bronchoscopic lung volume reduction appears to be safer than surgery and this enhanced safety profile presents an attractive alternative to COPD patients who are physiologically fragile. Efficacy data in the form of short-term, subjective improvement in dyspnoea and quality of life are readily available from small, nonrandomized studies. In contrast, minimal clinically important differences in objective endpoints such as spirometry and exercise capacity have not been a consistent finding. Refining patient selection to identify optimal candidates for each individual endoscopic modality is likely to improve outcomes in future. It is also hoped that the ongoing, larger randomized controlled trials will separate the therapeutic effect from the placebo effect of these procedures.

The VENT study was published at the time of this paper. This study randomized 220 patients with heterogeneous emphysema to receive duckbill endobronchial valves and 101 patients to standard medical care. At 6 months, there was a 6%-7% difference in FEV_1_ and 6-minute walk in favor of bronchoscopic lung volume reduction. However, short-term morbidity at 90 days was identified in the intervention arm with increases in COPD exacerbations and hemoptysis [35]. Patients and physicians will have to decide if this tradeoff is worthwhile whilst endoscopic techniques continue to be improved.

Although, bronchoscopic lung volume reduction still remains experimental and its benefits unproven, the data that is emerging holds much promise. Perhaps in future, these endoscopic modalities can even be used in combination with endobronchial valves targeting disease in heterogeneously diseased upper lobes while airway bypass reducing hyperinflation in more homogenously affected lower lobes [11]. Bronchoscopic therapy can also help wean patients off ventilators and may serve as a bridge to surgery or lung transplant [36]. The evolution of these ingenious techniques coupled with the accumulating clinical experience aim to improve endoscopic technology such that the majority of patients with severe COPD can soon be offered efficacious therapeutic options with far less risk of complications.

## Figures and Tables

**Table 1:** Bronchoscopic lung volume reduction modalities with their indications and commonly encountered complications.

Bronchoscopic lung volume reduction modality	Indications	Common complications
Endobronchial blockers	Heterogeneous Emphysema	(1) Blocker migration
		(2) Postobstructive pneumonia

Airway bypass stents	Homogenous Emphysema	(1) COPD exacerbation
		(2) Pneumonia/bronchitis
		(3) Air leak/pneumomediastinum

Endobronchial valves	Heterogeneous Emphysema	(1) COPD exacerbation
		(2) Pneumothorax
		(3) Bleeding
		(4) Pneumonia

Thermal vapor ablation	Heterogeneous Emphysema	(1) COPD exacerbation
		(2) Pneumonitis

Biological sealants	Both homogenous and heterogeneous emphysema	(1) COPD exacerbation
		(2) Pneumonia/aspiration

Airway implants/coils	Both homogenous and heterogeneous emphysema	Data not yet available
